# Correction to: Benchmarking the empirical accuracy of short-read sequencing across the *M. tuberculosis* genome

**DOI:** 10.1093/bioinformatics/btad107

**Published:** 2023-03-09

This is a correction to: Maximillian Marin, Roger Vargas, Jr, Michael Harris, Brendan Jeffrey, L Elaine Epperson, David Durbin, Michael Strong, Max Salfinger, Zamin Iqbal, Irada Akhundova, Sergo Vashakidze, Valeriu Crudu, Alex Rosenthal, Maha Reda Farhat, Benchmarking the empirical accuracy of short-read sequencing across the *M. tuberculosis* genome, *Bioinformatics*, Volume 38, Issue 7, March 2022, Pages 1781–1787, https://doi.org/10.1093/bioinformatics/btac023

In the originally published version of this manuscript, the orange and blue labels in Figure 3A were transposed.

This error has now been corrected online.

